# The RIG-I Signal Pathway Mediated *Panax notoginseng* Saponin Anti-Inflammatory Effect in Ischemia Stroke

**DOI:** 10.1155/2021/8878428

**Published:** 2021-08-20

**Authors:** Chujun Zhang, Sai Zhang, Lanxiang Wang, Soyeon Kang, Jiabao Ma, Shihao Liu, Yanhong Hu, Fan Zhang, Tianshi Sun, Yixin Dong, Tenghui Li, Weihong Li, Qinghong Du

**Affiliations:** ^1^School of Traditional Chinese Medicine, Beijing University of Chinese Medicine, Beijing, China; ^2^Nanchang Hongdu Hospital of Traditional Chinese Medicine, Nanchang, China; ^3^Department of Traditional Chinese Medicine, The 8th Medical Center of Chinese PLA General Hospital, Beijing, China; ^4^Department of Cell and Developmental Biology, School of Molecular and Cellular Biology, University of Illinois at Urbana-Champaign, USA; ^5^School of Nursing, Beijing University of Chinese Medicine, Beijing, China; ^6^Institute of Tibetan Medicine, University of Tibetan Medicine, Lhasa, China

## Abstract

*Panax notoginseng* saponins (PNS), the main bioactive constituents of a traditional Chinese herb *Panax notoginseng*, were commonly used for ischemic stroke in China. However, the associated cellular and molecular mechanisms of PNS have not been well examined. This study aimed to decipher the underlying molecular target of PNS in the treatment of cerebral ischemia. The oxygen-glucose-deprived (OGD) model of rat brain microvascular endothelial cells (BMECs) was used in this study. The alteration of gene expression in rat BMECs after PNS treatment was measured by microarray and indicated that there were 38 signaling pathways regulated by PNS. Among them, RIG-I receptor and related signaling molecules TNF receptor-associated factor 2 (Traf2) and nuclear factor-kappa B (NF-*κ*B) were significantly suppressed by PNS, which was verified again in OGD-induced BMECs measured by FQ-PCR and western blotting and in middle cerebral artery occlusion (MCAO) rats measured by immunohistochemistry. The levels of TNF-*α*, IL-8, and the downstream cytokines regulated by RIG-I receptor pathway were also decreased by PNS. Meanwhile, the neurological evaluation, hematoxylin and eosin (HE) staining, and Evans blue staining were conducted to evaluate the effect of PNS in MCAO rats. Results showed PNS significantly improved functional outcome and cerebral vascular leakage. Flow cytometry showed the number of the inflammatory cells infiltrated in brain tissue was decreased in PNS treatment. Our results identified that RIG-I signaling pathway mediated anti-inflammatory properties of PNS in cerebral ischemia, which provided the novel insights of PNS application in clinics.

## 1. Introduction

Ischemic cerebral apoplexy, which is more commonly known as stroke, is an acute condition compromise of the cerebral perfusion or vasculature or cerebrovascular accident and one of the leading causes of death worldwide. The initial ischemic injury can trigger a cascade of detrimental events including glutamate-associated excitotoxicity, inflammation, oxidant stress, and apoptosis [1, 2]. In recent years, more and more studies focused on examining the role of inflammation in the pathogenesis of cerebral ischemia. It has been known that ischemic stroke can induce acute inflammation in ischemic core and penumbra, which aggravates primary brain damage [3]. Inflammatory mediators secreted in ischemic lesion including cytokines, prostaglandins, free radicals, and chemokines can recruit other immune cells and participate in the already overactivated inflammatory responses and results in further damage of brain cells [4]. Thus, regulating certain inflammation-related pathways to reduce the release of inflammatory factors plays a role of great significance to improve brain damage.

Over the past few decades, remarkable advances have been made in understanding the basic molecular mechanisms of the stroke. However, the current therapeutic strategies for ischemic stroke are unsatisfactory and it is critical to discover better options in treatment. *Panax notoginseng* (San Qi in Chinese), the root of *Panax notoginseng* (Burk.) F. H. Chen of the Araliaceae family, is a traditional medicinal herb that has a long history of clinical application in Traditional Chinese Medicine since the Ming Dynasty according to the Ben Cao Gang Mu (Compendium of Materia Medica, 1596 A.D.) written by Shizhen Li (AD1518–1593). In ancient China, *Panax notoginseng* was traditionally used for the treatment of blood disorders and traumatic injuries, such as hemoptysis, metrorrhagia, or swelling due to blood stasis. In modern clinical applications, it had been found that notoginseng exerts various effects on the cardiovascular diseases [5], cerebrovascular diseases [6], cancers [7], etc. Pharmacochemical studies showed that *Panax notoginseng* contains various chemical components such as saponins, amino acids, polysaccharides, and flavonoids [8] with *Panax notoginseng* saponin (PNS) being the most important one. To date, twenty-seven saponins were identified in notoginseng, of which ginsenosides Rb_1_, Rg_1_, Re_1_, and Rh_1_ and notoginsenoside R_1_ were considered to be the major components of PNS. Recent studies suggested that PNS can increase blood flow and inhibit inflammation and platelet aggregation as well as attenuate oxidative stress [9, 10].

In China, the efficacy of purified PNS, as an injection, had been proved in clinical treatment of cerebral infarction. The pharmacological mechanism of PNS for improving ischemic injure has been extensively studied and some progress has been made. A recent meta-analysis study revealed that PNS may exert multiple protective mechanisms against ischemic-induced brain damage including hemostasis, anticoagulation, antithromboembolism, cerebral vasodilation, invigorated blood dynamics, anti-inflammation, antioxidation, and anti-hyperglycemic effects [11]. Many molecular pathways have been proposed to contribute to the pharmacology. However, the molecular target and signal pathway regulated by PNS have not been fully characterized.

Our previous studies suggested that PNS can regulate the paracrine secretion of brain microvascular endothelial cells (BMECs) in ischemic stroke [12]. This study aimed to find out the signal pathway regulated by PNS in BMECs and decipher the downstream mechanism of how PNS attenuate ischemic-associated injuries. Firstly, the microarray was used to screen the genes regulated by PNS in the oxygen-glucose deprivation- (OGD-) induced BMECs. Through the pathway analysis, we found that retinoic acid-inducible gene-I (RIG-I) reception signaling pathway was downregulated by PNS. Then, the change of RIG-I signaling pathway and the downstream effect was further verified in in vitro and vivo ischemic model. Our results suggested RIG-I signaling pathway mediated the anti-inflammatory effect of PNS on cerebral ischemia.

## 2. Materials and Methods

### 2.1. Materials

PNS is the total saponins extracted from the main roots or rhizomes of *Panax notoginseng* (Burk.) F. H. Chen. PNS used in this study was purchased from the National Institutes for Food and Drug Control (NIFDC, China), composed of notoginsenoside R_1_ 7.4%, ginsenoside Rg_1_ 26.3%, ginsenoside Re 3.7%, ginsenoside Rb_1_ 27.7%, and ginsenoside Rd 7.6%. Its HPLC analysis and chemical structure of each component are shown in the Supplementary Materials section.

RIG-I siRNA(R), siRNA Reagent System, was purchased from Santa Cruz Biotechnology (St Cruz, CA, USA). Lipopolysaccharide (LPS) was purchased from Sigma Aldrich (St. Louis, MO, USA). Rabbit antirat RIG-I, TNF receptor-associated factor 2 (Traf2), and nuclear factor-kappa B (NF-*κ*B) (p65) antibody were purchased from Cell Signaling Technology (Danvers, MA, USA). NF-*κ*Β DNA-binding enzyme-linked immunosorbent assay (ELISA) kit was purchased from Active Motif (Carlsbad, CA). ELISA kits for rat TNF-*α* and IL-8 were purchased from Cusabio (Houston, TX, USA).

### 2.2. Animals

50 Sprague-Dawley (SD) neonatal rats, weighing 20–25g, were used for BMEC culture. 40 male SD rats, weighing 260–280g, were used in ischemic animal model. All rats were obtained from Beijing Vital River Laboratory Animals Co., Ltd. (Beijing, China). Conformed to the National Institute of Health Guide for the Care and Use of Laboratory Animals (NIH Publications No. 80–23), all animal procedures were taken to minimize animal discomfort and reduce the number of animals used, and all experiments were approved by the Animal Experimental Ethical Committee of Beijing University of Chinese Medicine.

### 2.3. Preparation of Rat BMECs and OGD Model

Detailed protocol of isolation and culture of rat BMECs was introduced in our previous study [12]. In brief, the cerebral cortex of neonatal rats was isolated and minced into small pieces. The brain gray matter was homogenized and filtered through 180°*μ*m and 75°*μ*m mesh sequentially. The fragments on the 75 *µ*m mesh were collected and digested with 0.2% type II collagenase at 37°C for 20 min. Finally, the microvessel segments were collected and seeded in gelatin-coated dishes. The 3rd passage cells (purity 98%) were used for the experiments.

The OGD model of rat BMECs was established according to the method introduced in our previous study [12]. Briefly, the BMECs were cultured with sugar-free Krebs solution (117.0 mM NaCl, 4.7 mM KCl, 25 mM NaHCO_3_, 1.2 mM KH_2_PO_4_, 2.5 mM CaCl_2_, 1.2 mM MgCl_2_, pH 7.2–7.4) in 95% N_2_ and 5% CO_2_ environment for 6 h.

### 2.4. Microarray Analysis

#### 2.4.1. Groups and Drug Administration of BMECs

The BMECs were divided into two groups: OGD group and OGD + PNS group. Cells in OGD + PNS group were treated with 22 *μ*g/ml PNS (which was the optimal concentration of PNS determined in a previous study) in the modeling process.

#### 2.4.2. Gene Chip Array

RNA samples were extracted following manufacturers' instruction and purified with RNeasy Mini Kit (QIAGEN, Germany). Total RNA (500 ng) was reversely transcribed into cDNA with Ambion® WT Expression Kit (Ambion, USA) according to the Affymetrix GeneChip Expression Analysis instruction. The labeled cDNA was hybridized on Affymetrix GeneChip Rat Gene 1.0 ST Array for 16 h at 45°C. The gene processing was performed using Fluidics Station 450 and the gene chip was scanned by Scanner 3000 7G. Then, microarray expression data were analyzed by applying a series of quality control, statistical, filtering, and algorithms.

#### 2.4.3. Pathway Analysis

Based on the Kyoto Encyclopedia of Genes and Genomes (KEGG) and BioCarta database, pathway analysis was conducted to find out the significant pathway from the differential genes between two groups by Fisher's exact test and chi-square test; the threshold of significance was defined by *p* value and FDR.

The results of pathway analysis showed that 38 biological pathways were regulated by PNS in OGD-induced BMECs. Among them, RIG-I-like receptor signaling pathway attracted our interest. Therefore, the following experiments were designed to verify the RIG-I signaling-mediated anti-inflammatory properties of PNS in in vitro and in vivo mode.

### 2.5. Effect of PNS on RIG-I Signaling Pathway in Injured BMECs

#### 2.5.1. Groups and Drug Administration of BMECs

Two experiments were carried out to examine the effect of PNS on RIG-I signaling pathway. Firstly, OGD model was established as mentioned above and short interfering RNA was used to silence the expression of RIG-I as controls. BMECs were randomly divided into four groups: (1) normal group; (2) OGD model group; (3) PNS group: the OGD-injured BMECs treated with PNS; and (4) RIG-I siRNA group: RIG-I siRNA was transfected into BMECs before modeling. Details of transfection protocol and efficiency are shown in the Supplementary Materials section.

LPS was known as an activator of RIG-I signaling pathway. To further examine the specific effect of PNS on RIG-I signaling pathway, we applied LPS-induced activated model by exposing BMECs to 1 *μ*g/ml LPS [13]. The effect of PNS on RIG-I signaling pathway in LPS-induced BMECs was investigated. The BMECs were randomly divided into three groups: (1) normal group; (2) LPS group: BMECs exposed to 1 *μ*g/ml LPS; and (3) LPS + PNS group: LPS-induced BMECs treated with 22 *μ*g/ml PNS.

#### 2.5.2. Fluorogenic Quantitative PCR (FQ-PCR)

Total RNA was extracted from BMECs using TRIzol Reagent (Invitrogen) according to the manufacturer's instruction. 2 *μ*g total RNA was used for reverse transcription. FQ-PCR was performed to detect RIG-I, Traf2, and NF-*κ*B (p65) mRNA expression on a ABI 7500 Real-Time PCR System (Bio-Rad, USA) using the protocol as follows: 40 circles as predenaturation at 95°C for 15 s, renaturation at 60°C for 30 s, and extension at 72°C for 15 s. The multiple change of relative gene expression was calculated using 2^−ΔΔCT^.The primers used were as follows: RIG-I for GAAATACAACGACGCCCTCA, Rev： AAATGCTGCTTCTCGGACAT; Traf2 for GGGACAAGGTTAGAAGCCAAG, Rev: CAGGCAGAAGGAGCAGTAGC; NF-*κ*B for TGGGACGACACCTCTACACA, Rev: GGCTCAAAGTTCTCCACCAG; *β*-actin for TATCGGCAATGAGCGGTTCC, Rev: AGCACTGTGTTGGCATAGAGG.

#### 2.5.3. Western Blotting

RIG-I, Traf2, and NF-*κ*B (p65) protein expressions were detected by western blotting as previously described [14]. In brief, BMECs were harvested in lysis buffer and protein extract was collected and quantified by BCA protein assay reagent kit. The protein was separated by SDS-PAGE on 10% polyacrylamide gels and transferred to a polyvinylidene difluoride (PVDF) membrane. The membranes were blocked for 1.5 h in 5% nonfat milk powder solution before incubated with rabbit anti-rat monoclonal RIG-I antibody (1 : 1000), Traf2 antibody (1 : 1000), and NF-*κ*B (p65) antibody (1 : 1000) overnight at 4°C. After 3 rinses, the membranes were incubated at RT for 2°h with appropriate secondary antibody (1 : 5000). *β*-Actin was used as an internal reference. Bands were visualized with a chemiluminescence substrate system ECL.

#### 2.5.4. Enzyme-Linked Immunosorbent Assay (ELISA)

Nuclear protein of cells in each group was extracted by nuclear extraction kit. NF-*κ*Β p65 DNA binding activity was identified by TransAM NF-*κ*Β DNA-binding ELISA kit following the manufacturer's instructions. The TNF-*α* and IL-8 contents in BMECs and brain tissue of rats were measured using commercial rat TNF-*α* ELISA kit and IL-8 ELISA kit.

### 2.6. Effect of PNS on RIG-I Signaling Pathway in Rat Model of Focal Cerebral Ischemia

#### 2.6.1. Middle Cerebral Artery Occlusion (MCAO) Model of Rats

A permanent middle cerebral artery occlusion model of rats was established as introduced in detail in a previous study [15]. Briefly, rats were anesthetized with 10% hydrate chloral solution. A heat-blunted nylon suture was inserted into the right external carotid artery and advanced until it obstructed the MCA together with the ligation of the common carotid artery. After lesion closure, the suture was permanently retained. Following MCAO, rats were placed in temperature-controlled recovery cages for 2 h to prevent postsurgery hypothermia.

#### 2.6.2. Groups and Drug Administration of the Rats

40 male SD rats were randomly assigned to three groups using table of random number: sham group (*n* = 10), model group (*n* = 15), and PNS group (*n* = 15). The latter two groups were subjected to the occlusion of right middle cerebral artery according to the above method. 8 rats died during or after operation were excluded from subsequent analyses. Animals in the sham-operated group underwent surgery but did not have the suture inserted. The rats in PNS group received intraperitoneal injection with PNS at 16.5 mg·kg^−1^ body weight (the dose of PNS was based on our previous experiments) at 48 h and 24 h before surgery. Control animals received an equal volume of saline at the same time point.

#### 2.6.3. Neurological Evaluation

All animals received a series of neurological functional assessments including left forelimb movement, muscle tension of both limbs, resistance to left and right retraction, and eye changes at 2 h, 24 h, and 72 h after stroke onset according to Bederson et al. [16]. The total score of the above four categories greater than 3 points was regarded as successful modeling.

#### 2.6.4. Hematoxylin and Eosin (HE) Staining

The brains of rats were removed and fixed overnight in 4% paraformaldehyde solution. Brain biopsies were dehydrated through a serial alcohol gradient and embedded in paraffin wax block. Brain samples were cut into coronal sections (6 *µ*m thickness) using a cryostat. The sections were stained with hematoxylin and eosin. After staining, the sections were dehydrated with graded alcohol and xylene.

#### 2.6.5. Evans Blue (EB) Staining

EB staining was used to measure the damage of endothelium in MCAO rats. EB (2%) was intravenously injected at 2 h before brain collection. Rats were transcardially perfused with saline. Then, brains were taken out and cut into thick slices. The ipsilateral forebrain was homogenized and diluted with trichloroacetic acid. After centrifuged, the supernatant was collected and the absorbance of supernatant was detected at 620 nm by using a spectrophotometer. The amount of EB (*μ*g/g) was calculated from a standard curve.

#### 2.6.6. Immunohistochemistry Staining

Immunohistochemistry investigation was performed to examine the expression of RIG-I, Traf2, and NF-*κ*B (p65) in MCAO model. The brain tissues were fixed with 4% paraformaldehyde for 2 days, embedded in paraffin, and then sectioned into 7 *μ*m thick slices. The slices were treated with 3% H_2_O_2_ for 10 minutes before incubated with a rabbit anti-rat monoclonal RIG-I、Traf2, or NF-*κ*B (p65) antibody (1 : 200) overnight at 4°C. After rinsed three times in PBS, the slices were incubated with corresponding secondary antibodies for 30 minutes at 37°C. The slides were visualized with DAB kit (ZSGB-BIO, China) and imaged by using a microscope (OLYMPUS, Japan).

#### 2.6.7. Flow Cytometry

The mice were used and subjected to MCAO in the part of experiment. The brain tissues of mice were taken out and fully homogenized and successively added 30% and 70% Percoll (GE Healthcare, USA) and then centrifuged at room temperature for 30 minutes. The cells between 30% and 70% Percoll were collected and added Cell-ID Cisplatin Stain (Fluidigm), centrifuged again at 300g for 5 minutes, and then stained with using combinations of antibodies against mouse CD45 (BD Pharmingen, USA), CD11b (BD Pharmingen, USA), and Ly6G (BD Pharmingen, USA) for 1 h at room temperature in the dark. The FlowJo software (Tree Star) was used to analyze. The data are presented as a percentage of CD11b^+^ and CD11b^+^ Ly-6G^+^ cells in total CD45^+^ cells.

### 2.7. Statistical Analyses

All data in this study were presented as mean ± SD. Gene chip analysis was performed using the Affymetrix GeneChip Operating Software (Gminix, China); two-side Fisher's exact test and *χ*^2^ test were used to select the significant pathway (*p* value ≤ 0.05). Other measurement data were analyzed by SPSS 22.0 statistical software. First, a test of normality and homogeneity of variance was performed. Data between two groups obeying normal distribution and homogeneous variance were evaluated by one-way analysis of variance (ANOVA). Otherwise, nonparametric test was used. *p* ≤ 0.05 was considered statistically significant. Correlations were analyzed by Pearson test and the correlation coefficients (*r*) were calculated.

## 3. Results

### 3.1. 38 Signaling Pathways Regulated by PNS Were Screened Out by Genomic Analysis

The differentially expressed genes in OGD-induced BMECs treated with or without PNS were screened by Affymetrix GeneChip Array. The microarray results showed there were 1195 differentially expressed genes in the PNS group compared to BMECs of the OGD group, including 552 upregulated genes and 643 downregulated genes. The most significantly affected genes included those coding for proteins involved in oxidation-reduction process, transmembrane transport, ion transport, and immune response.

**Figure 1:**
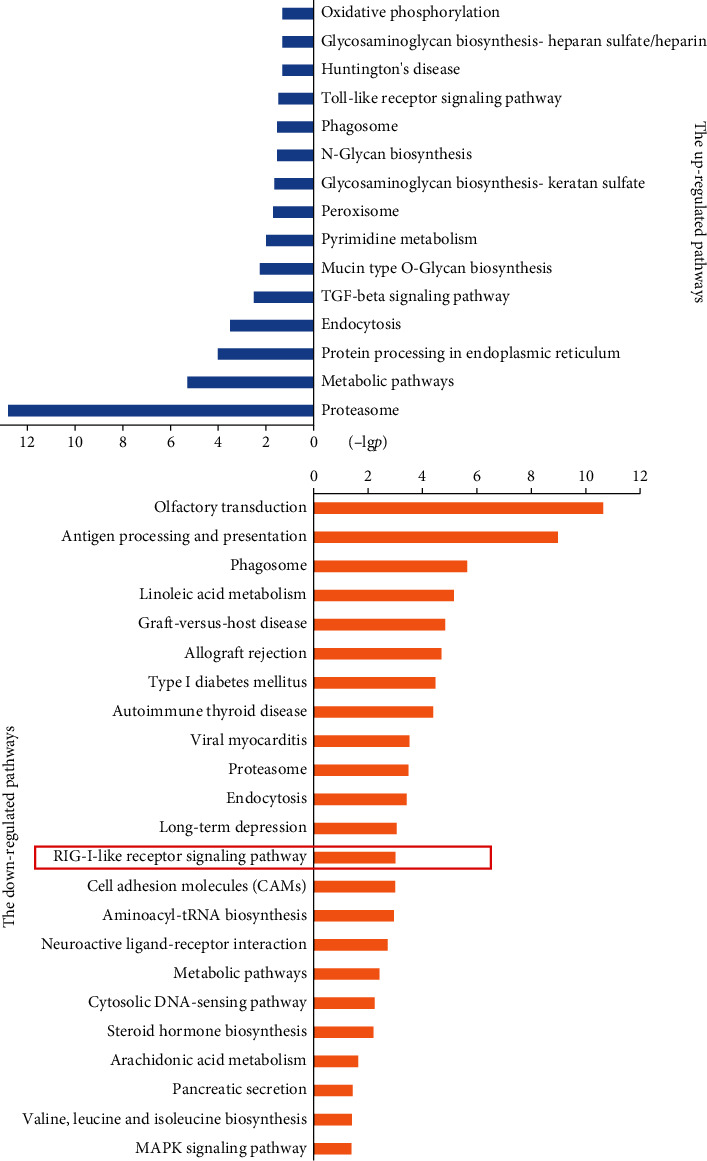
The signaling pathways regulated by PNS in OGD-induced BMECs. Based on genomic analysis, 15 biological pathways were upregulated and 23 pathways were downregulated by PNS in OGD-induced BMECs. The RIG-I-like receptor signaling pathway was downregulated by PNS.

Next, we analyzed the signal pathways involved in the 1195 differential genes detected by the gene chip through pathway analysis tools based on KEGG. The results showed 15 upregulated pathways and 23 downregulated pathways in OGD-induced BMECs treated with PNS (Figure 1). The PNS-affected pathways mainly contained olfactory transduction, metabolic pathways, TGF-beta signaling pathway, proteasome, and so on. Among them, RIG-I-like receptor signaling pathway attracted our attention the most. RIG-I is traditionally considered as an intracellular molecule that responds to viral nucleic acids and triggers antiviral innate immunity. Recent studies indicated a role for RIG-I in managing inflammatory response after focal cerebral ischemia [15, 17]. In this experiment, three genes involved in RIG-I signaling pathway, namely, Ddx58/RIG-I, Traf2, and Rel A/NF-*κ*B (p65) were downregulated by PNS (Table 1). Therefore, the expressions of three genes were further confirmed in the following experiments.

### 3.2. RIG-I Signaling Pathway Was Downregulated by PNS in OGD-Induced BMECs

**Figure 2:**
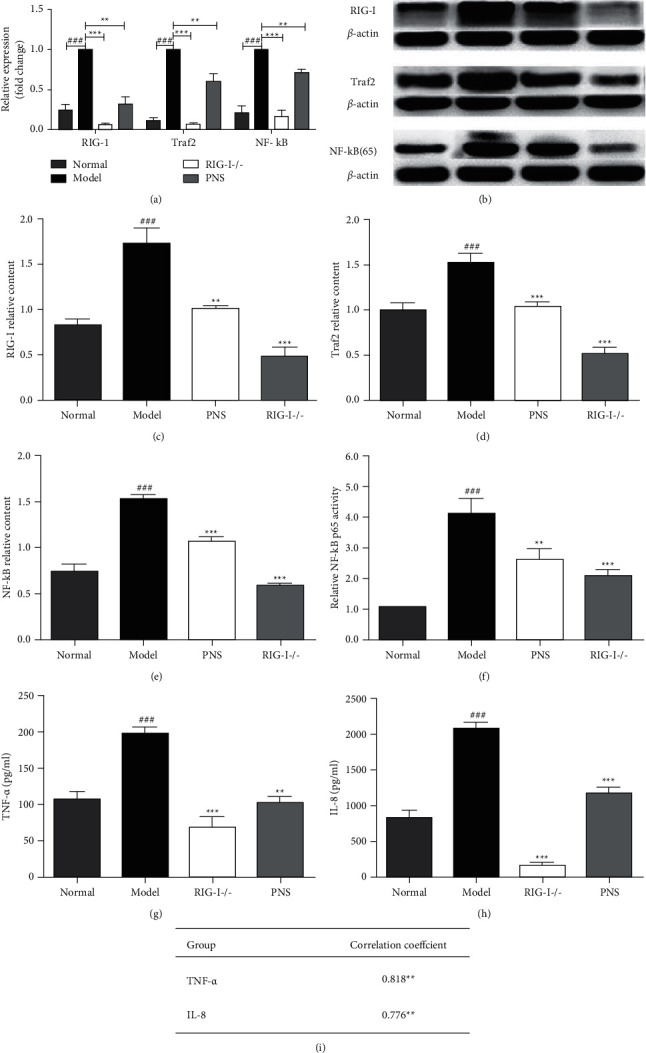
The effect of PNS on RIG signaling pathway and downstream inflammatory factors in OGD-induced BMECs. The OGD-injured BMECs were administrated with 22 *μ*g/ml PNS. siRNA was used to silence RIG-I gene as control. (a) The mRNA levels of RIG-I, Traf2, and NF-*κ*B (p65) were detected by RQ-PCR. (b) The expressions of RIG-I, Traf2, and NF-*κ*B (p65) were detected by western blotting. Quantitative analysis of RIG-I (c), Traf2 (d), and NF-*κ*B (p65) (e) protein levels are shown. (f) The result of p65 DNA binding assay. The content of TNF-*α* (g) and IL-8 (h) was detected by ELISA. (i) Analysis of the correlation showed that production of TNF-*α*, IL-8, and RIG-I signaling pathway was significantly correlated. Data are presented as mean ± s.e.m. ^###^*p* < 0.001 versus normal group,  ^*∗∗*^*p* < 0.01,  ^*∗∗∗*^*p* < 0.001 versus model group.

As suggested in Figure 2, the mRNA and protein expressions of RIG-I, Traf2, and NF-*κ*B (p65) in the model group were significantly higher than that in the normal group (*p* < 0.001), indicating that OGD induced the activation of RIG-I signaling pathway. p65 DNA binding assay (Figure 2(f)) demonstrates significantly increased nuclear translocation and DNA binding activity of NF-*κ*B in the OGD group. This increase was abolished after the knockdown of RIG-I, indicating that Traf2 and NF-*κ*B were the downstream signal molecules of RIG-I pathway. The expressions of RIG-I, Traf2, and NF-*κ*B (p65) and activity of nuclear NF-*κ*B in the PNS group was significantly decreased compared to the model group (*p* < 0.01), suggesting that PNS suppressed the RIG-I signaling pathway and showed a similar effect with RIG-I siRNA.

### 3.3. PNS Reduced Production of IL-8 and TNF-*α* in OGD-Injured BMECs

As the graph of RIG-I signaling pathway in KEGG database, RIG-I could activate NF-*κ*B (p65) via Traf2, which affects the transcription of many inflammatory factors. The IL-8 and TNF-*α* are main downstream factors of RIG-I signaling pathway. Sequentially, the IL-8 and TNF-*α* contents in BMECs were measured by ELISA. As shown in Figures 2(g) and 2(h), the levels of TNF-*α* and IL-8 in the OGD group were significantly higher than the normal group (*p* < 0.001), which was abolished by RIG-I siRNA. Meanwhile, the correlation analysis showed the correlation coefficient of TNF-*α* and RIG-I was 0.818; the correlation coefficient between IL-8 and RIG-I was 0.776 (Figure 2(i)). The results further confirmed that production of TNF-*α* and IL-8 was related to activity of RIG-I signaling pathway. Compared with the OGD group, TNF-*α* and IL-8 levels in the PNS groups were significantly decreased (*p* < 0.01), suggesting that PNS inhibited production of TNF-*α* and IL-8, which involved in the depression of RIG-I signaling pathway.

### 3.4. PNS Downregulated the RIG-I Signaling Pathway in LPS-Induced BMECs

**Figure 3:**
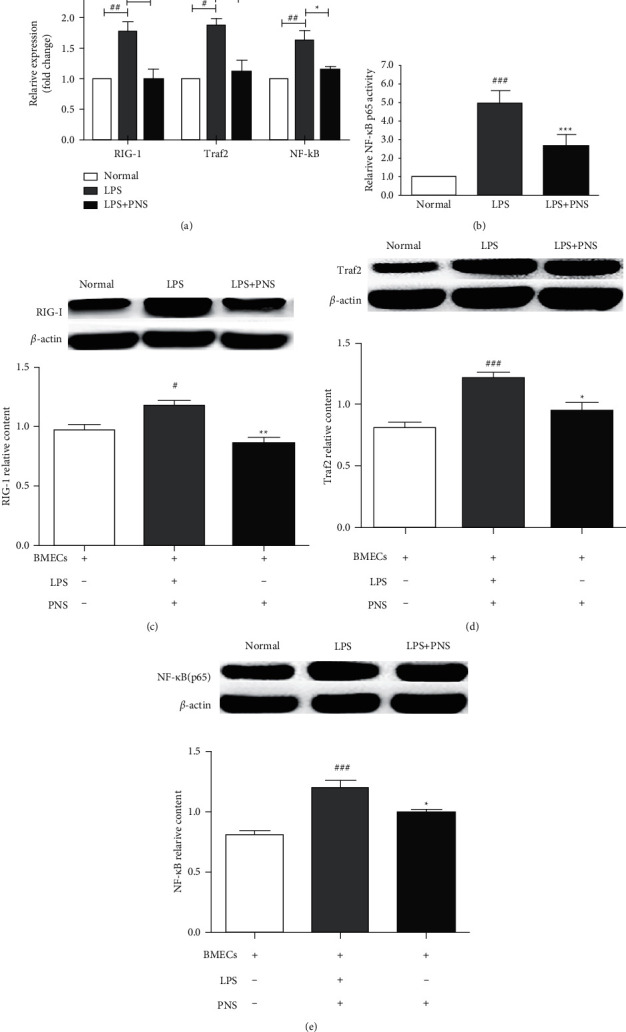
The effect of PNS on RIG-I/Traf2/NF-*κ*B (p65) signaling pathway in LPS-induced BMECs. (a) Bar graphs showed the mRNA levels of RIG-I, Traf2 and NF-*κ*B examined by RQ-PCR. (b) Bar graph showed the result of p65 DNA binding assay. (c) (d) (e) showed the protein levels of RIG-I, Traf2 and NF-*κ*B (p65) measured by Western boltting. Data were presented as mean ± s.e.m. ^#^*p* < 0.05, ^##^*p* < 0.01, ^###^*p* < 0.001versus normal group; *∗p* < 0.05,  ^*∗∗*^*p* < 0.01 versus LPS group.

To further confirm RIG-I signaling pathway as the molecular target of PNS, we adopted LPS, as an activator, to activate RIG-I signal and evaluated the regulating effect of PNS on RIG-I signaling pathway. Interesting, PNS suppression was also found in LPS-stimulated BMECs. As shown in Figure 3, the expression of RIG-I, Traf2, NF-*κ*B (p65) and activity of nuclear NF-*κ*B in BMECs exposed to 1 *μ*g/ml LPS were significantly increased (*p* < 0.05, *p* < 0.01, *p* < 0.001) and were decreased after PNS treatment (*p* < 0.05, *p* < 0.01).

### 3.5. Protective Effect of PNS in MCAO Model

**Figure 4:**
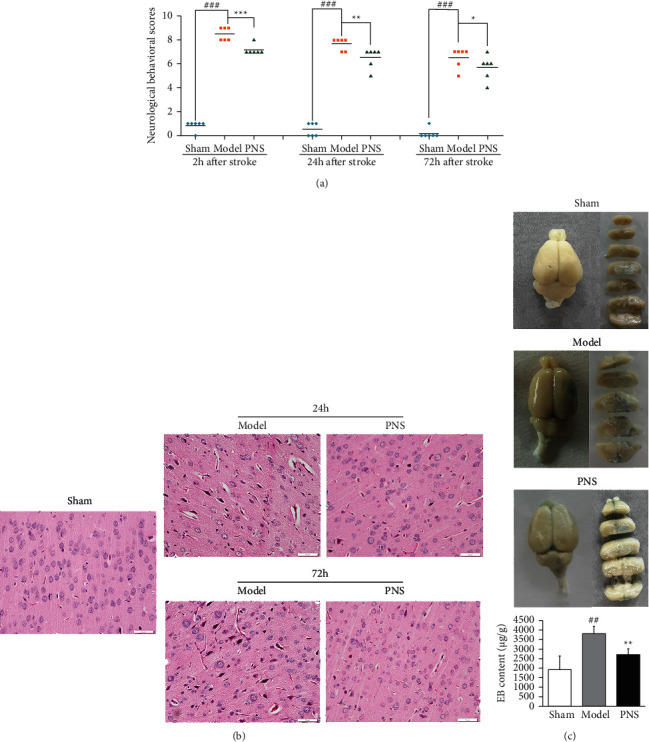
The Protective effect of PNS on ischemic brain injury in MCAO rats. (a) Neurological behavioral evaluation was operated at 2h, 24h and 72h after stroke onset. (b) HE staining was examined at 24h and 72h after stroke onset. (c) Evans blue staining was used to indicate the impair of endothelium in MCAO rats. ^##^*p* < 0.01, ^###^*p* < 0.001 versus sham group;  ^*∗∗∗*^*p* < 0.001,  ^*∗∗*^*p* < 0.01, *∗p* < 0.05 versus model group.

The effect of PNS was further proved in MCAO rat model. Neurological behavioral evaluation was operated at 2h, 24h, 72h after stroke onset.  Figure 4(a) showed that the subjects in the PNS treatment group exhibited less severe neurological deficits compared with the rats in model group at various time-points (*p* < 0.001, *p* < 0.01, *p* < 0.05), suggesting treatment of PNS significantly improved functional outcome.

Histological examination (Figure 4(b)) showed that the structure of the brain tissue and neurons was normal and intact in the sham group. MCAO 24 hours later, some neurons were necrotic, accompanied by inflammatory cell infiltration and edema around neurons and blood vessels. The vacuolar softening structure appeared in the intercellular substance. 72 hours after the model, severe nuclear pyknosis occurred, and the nuclear staining became deeper, suggesting that more neurons were necrotic. The edema of cells and tissues was more obvious. However, these ischemic morphological phenotypes including cellular swelling, nucleus atrophy, and bubble-like intercellular substances were ameliorated in PNS group.

EB content (Figure 4(c)) can reflect the damage of endothelium and vascular permeability. After 24-h ischemia, EB content in brain tissue was significantly raised compared with sham group (*p* < 0.01), while PNS significantly reduced the EB content (*p* < 0.01).

### 3.6. PNS Downregulated the RIG-I Signaling Pathway and Alleviated Inflammation in MCAO Rats

**Figure 5:**
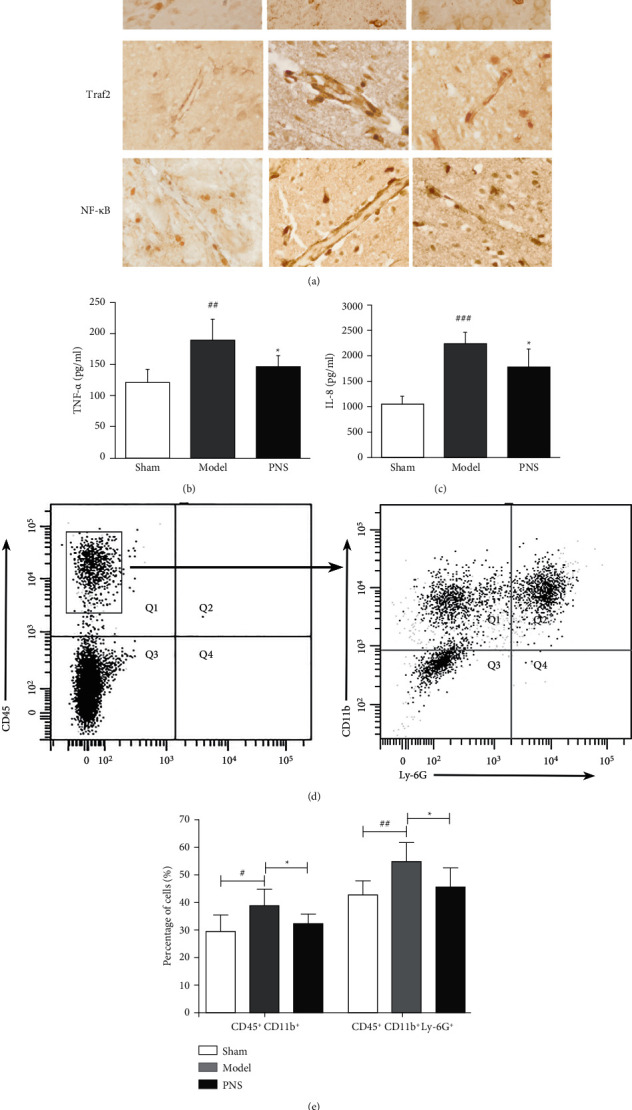
The effect of PNS on expression of RIG-signaling pathway and inflammatory cell infiltration in MCAO rats. (a) The expression of RIG-I, Traf2, NF-*κ*B (p65) were detected in vascular endothelium of MCAO rats by immunohistochemical experiment (magnification, ×1000). The levels of TNF-*α* (b) and IL-8 (c) were measured by ELISA. (d) Showed the gating strategy for myeloid cells using the surface markers CD45, CD11b and Ly6G by flow cytometry. Bar graph (e) showed the percentage of myeloid cells subsets. ^##^*p* < 0.01, ^###^*p* < 0.001 versus sham group; *∗p* < 0.05 versus model group.

The immunohistochemical experiment was performed to determine the expression of RIG-I, Traf2 and NF-*κ*B (p65) in vascular endothelium after stroke. Results showed (Figure 5(a)) that the expressions of RIG-I, Traf2 and NF-*κ*B (p65) were almost invisible in the endothelial cells of the sham group, whereas the expression was increased in the model group and significantly reduced after PNS treatment.

As shown in Figures 5(b) and 5(c)), TNF-*α* and IL-8 content of the brain homogenate in model group were significantly higher than the normal group (*p* < 0.01), indicating that MCAO induced expression of inflammatory factor TNF-*α* and IL-8. Compared with the model group, TNF-*α* and IL-8 levels in the PNS groups were significantly decreased (*p* < 0.05).

The flow cytometry was used to detect inflammatory cell infiltration in the mouse brain tissue. As shown in Figure 5(d), Leukocyte population was first identified by expression of CD45^+^. The neutrophils (CD11b^+^ Ly6G^+^) were distinguished from total myeloid cells (CD11b^+^). Bar graphs (Figure 5(e)) depicted abundance of myeloid cells subsets. There was a greater inflammatory cell infiltration in brain tissue after stroke, whereas percentage of inflammatory cells was significantly reduced after PNS treatment (*p* < 0.05).

## 4. Discussion

RIG-I, an important member belong to RLRs family, is an innate immune receptor in the cytoplasm and induces anti-vital responses by specifically recognizing viral duplex RNA. Increasing evidences suggest that RIG-I is also involved in non-viral infectious inflammatory processes such as atherosclerosis, rheumatoid arthritis, cancers [18, 19]. Brand F et al [15] reported that the RIG-I protein expression increased in hippocampus after MCAO and involved in the inflammatory response. The regulatory functions of RIG-I are strikingly comprehensive and could activate a series of downstream signaling molecules including virus induced signaling adaptor (VISA), CARD adaptor inducing IFN-b (Cardif), interferon regulatory factor (IRF3) and NF-*κ*B, resulting in production of proinflammatory cytokines and chemokines [20, 21]. Therefore, the suppression of RIG-I signaling may be a potential target to block the inflammatory cascade often observed in stroke.

In this study, we discovered that genes in the RIG-I pathway including Ddx58/RIG-I, Traf2 and Rel A/NF-*κ*B(p65) were down-regulated by PNS in OGD-induced BMECs using the technique of gene chip and data analysis. The effect of PNS on RIG-I signaling pathway was further verified in in vitro and vivo ischemic models. Our results showed RIG-I expression levels increased both in OGD-induced BMECs and in the brain of MCAO rats and could be abolished by PNS treatment, suggesting RIG-I signaling pathway might be the potential therapeutic target of PNS. In addition, we discovered that PNS could reduce the increased expression of RIG-I, Traf2, NF-*κ*B (p65) after LPS activation, which further confirmed that PNS had a prominent effect on the regulation of RIG-I signaling pathway. The results of cellular experiments were validated in immunohistochemical experiment which suggested that PNS could reduce the expression of above parameter in the vessel wall of the MCAO rats.

TNF-*α* and IL-8 are two crucial inflammatory factors that involved in the ischemic cascade. Evidence suggests that TNF-*α* can induce brain damage by activating inflammatory cells, facilitating adhesion and disrupting the blood-brain barrier during ischemic attack [22, 23]. IL-8 is a potent neutrophil chemoattractant and activator. By leading to neutrophil accumulation, IL-8 gives rise to the secretion of toxic oxygen radicals and enzymes from neutrophils such as protease, gelatinize, and collagenase, which causes damage in the tissue [24]. According to the KEGG database, IL-8 and TNF-*α* are main downstream molecules in RIG-I signal pathway after activation of NF-*κ*B via Traf2. In this study, we used siRNA transfection to silence RIG‐I gene and found RIG-I siRNA led to decrease in production of TNF-*α* and IL-8, suggesting RIG-I could activate NF-*κ*B through Traf2 and affected the transcription of TNF-*α* and IL-8. We found PNS could effectively decrease the expression of TNF-*α* and IL-8 in ischemic endothelial cells and brain tissue. The correlation analysis showed that the secretion of TNF-*α* and IL-8 were associated with RIG-I expression. These results revealed that the effect of PNS on TNF-*α* and IL-8 secretion might be related to down-regulation of RIG-I signaling pathway.

It had been reported that PNS exerted extensively beneficial effects on cerebrovascular disease, especially ischemic stroke. PNS may ameliorate learning, memory deficits and blood viscosity by protecting neurons from oxidative stress in ischemic brain [25] and protect ischemic injured brain cells via activation of PI3K/Akt and Nrf2 signaling pathways [26]. Our results showed that PNS effectively improved the neurological function of MCAO rats and ameliorated the pathological changes associated to ischemic damage. In addition, it also significantly improved the cerebral vascular leakage, as indicated by EB extravasation assay. The pharmacologic action of PNS, as a complex of various saponins, is complicated. Anti-inflammatory effect was one of the crucial pharmacological mechanism of PNS in cerebrovascular disease. Previous study reported that PNS could reduce Ca^2+^ level in neutrophils to inhibit PLA2 activity and inhibit the production of inflammatory cytokine [27]. In this study, we used the flow cytometry to find that percentage of myeloid cells and neutrophils in the brain tissue were down-regulated by PNS, indicating PNS inhibited the infiltration of inflammatory cells in ischemic brain. The results were consistent with inhibiting release of IL-8 and TNF-*α* and the cerebral vascular leakage.

## 5. Conclusions

Our study suggested the RIG-I signaling pathway, as a novel signaling target of PNS, mediated anti-inflammatory properties of PNS in cerebral ischemia, which supported the use of PNS in clinic for anti-inflammation in ischemic diseases. However, whether the PNS regulates the signal pathway by prohibiting the specific structure of RIG-I is not clear. Thus, further investigations are needed to uncover the genetic mechanisms involved.

## Figures and Tables

**Table 1:** Differentially expressed genes involved in RIG-I signaling pathway.

Pathway ID	Pathway name	Gene name	*p* value	FDR	Enrichment
4622	RIG-I-like receptor signaling pathway	Ddx58	0.0010167	0.005997	7.6358723
4622	RIG-I-like receptor signaling pathway	Traf2	0.0010167	0.005997	7.6358723
4622	RIG-I-like receptor signaling pathway	Rel A	0.0010167	0.005997	7.6358723

## Data Availability

The data used to support the findings of this study are available from the corresponding author upon request.

## Abbreviations:

PNS: Panax notoginseng saponins
RIG-I: Retinoic acid-inducible gene-I
BMECs: Brain microvascular endothelial cells
LPS: Lipopolysaccharide
MCAO: Middle cerebral artery occlusion
OGD: Oxygen and glucose deprivation
Traf2: Tumour necrosis factor receptor-associated factor2
NF-*κ*B: Nuclear factor-kappa B.

## Supplementary Materials

Supplementary Materials 1. The chemical identification of *Panax notoginseng* saponins. *Panax notoginseng* saponins (PNS)is the total saponins extracted from the main roots or rhizomes of *Panax notoginseng* (Burk.) F. H. Chen. PNS used in this study was a standard substance that purchased from the National Institutes for food and drug Control (NIFDC, China), which was identifiably composed of Notoginsenoside R1, ginsenoside Rg1, ginsenoside Re, ginsenoside Rb1, ginsenoside Rd by HPLC analysis (Fig S1). The chemical structure of each component was shown (Fig S2). 2. Transfection of siRNAs in BMECs. Short interfering RNA was used to silence RIG-I as the control, to further determine the pivotal role of RIG-I in ischemic injury. Transfection was performed by culturing the BMECs in siRNA Transfection Medium containing the siRNA Transfection Reagent mixture according to the manufacturer's protocol. Blank siRNA was used as a control. The expression of RIG-I was detected by Western Blotting to evaluate the efficiency of the transfection. As shown in Figure S3, the expression of RIG-I protein in the RIG-I siRNA transfection was significantly lower than control group (*p* < 0.01), suggesting that the transfection was successful and efficient.
