# Supplementary figures and images for: The RIG-I Signal Pathway Mediated Panax notoginseng Saponin Anti-Inflammatory Effect in Ischemia Stroke
Source: Evid Based Complement Alternat Med. 2021 Aug 20;2021:8878428. doi: 10.1155/2021/8878428 (PMC8403041; doi:10.1155/2021/8878428)

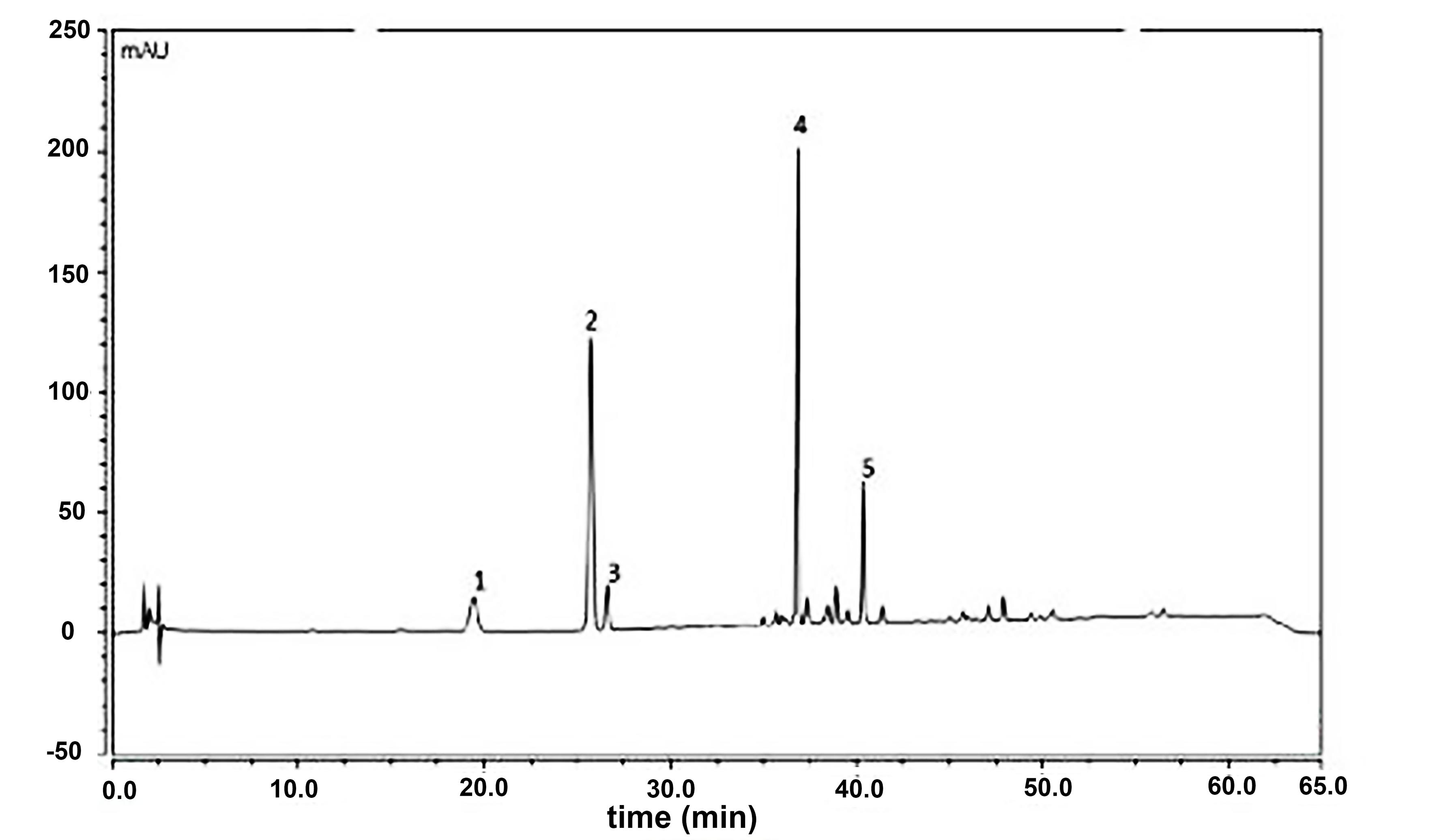

Supplement: Supplementary Materials — 1. The chemical identification of Panax notoginseng saponins. Panax notoginseng saponins (PNS)is the total saponins extracted from the main roots or rhizomes of Panax notoginseng (Burk.) F. H. Chen. PNS used in this study was a standard substance that purchased from the National Institutes for food and drug Control (NIFDC, China), which was identifiably composed of Notoginsenoside R1, ginsenoside Rg1, ginsenoside Re, ginsenoside Rb1, ginsenoside Rd by HPLC analysis (Fig S1). The chemical structure of each component was shown (Fig S2). 2. Transfection of siRNAs in BMECs. Short interfering RNA was used to silence RIG-I as the control, to further determine the pivotal role of RIG-I in ischemic injury. Transfection was performed by culturing the BMECs in siRNA Transfection Medium containing the siRNA Transfection Reagent mixture according to the manufacturer's protocol. Blank siRNA was used as a control. The expression of RIG-I was detected by Western Blotting to evaluate the efficiency of the transfection. As shown in Figure S3, the expression of RIG-I protein in the RIG-I siRNA transfection was significantly lower than control group (p < 0.01), suggesting that the transfection was successful and efficient. [file 8878428.f1.zip › figure s1.tif]

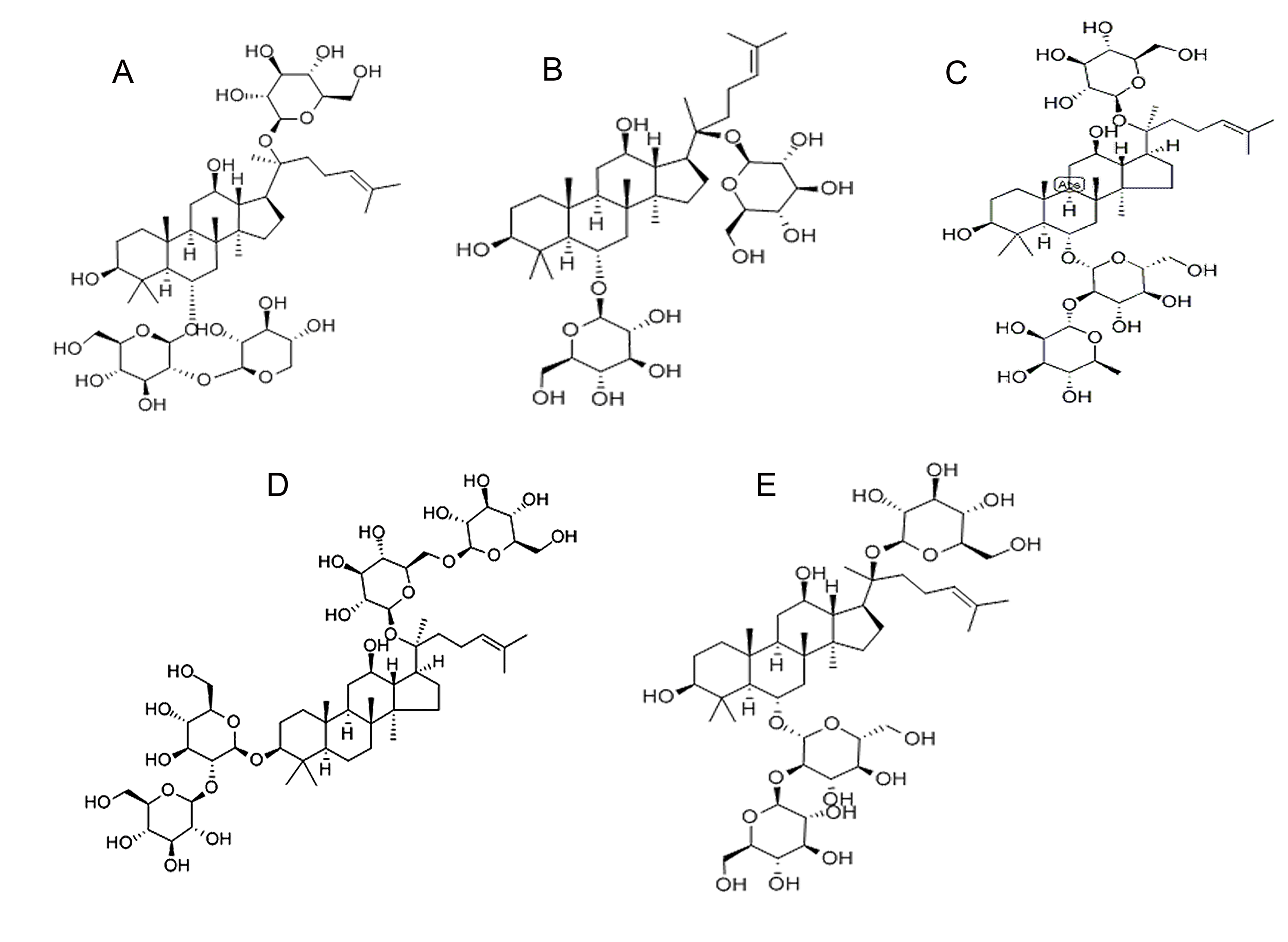

Supplement: Supplementary Materials — 1. The chemical identification of Panax notoginseng saponins. Panax notoginseng saponins (PNS)is the total saponins extracted from the main roots or rhizomes of Panax notoginseng (Burk.) F. H. Chen. PNS used in this study was a standard substance that purchased from the National Institutes for food and drug Control (NIFDC, China), which was identifiably composed of Notoginsenoside R1, ginsenoside Rg1, ginsenoside Re, ginsenoside Rb1, ginsenoside Rd by HPLC analysis (Fig S1). The chemical structure of each component was shown (Fig S2). 2. Transfection of siRNAs in BMECs. Short interfering RNA was used to silence RIG-I as the control, to further determine the pivotal role of RIG-I in ischemic injury. Transfection was performed by culturing the BMECs in siRNA Transfection Medium containing the siRNA Transfection Reagent mixture according to the manufacturer's protocol. Blank siRNA was used as a control. The expression of RIG-I was detected by Western Blotting to evaluate the efficiency of the transfection. As shown in Figure S3, the expression of RIG-I protein in the RIG-I siRNA transfection was significantly lower than control group (p < 0.01), suggesting that the transfection was successful and efficient. [file 8878428.f1.zip › figure s2.tif]

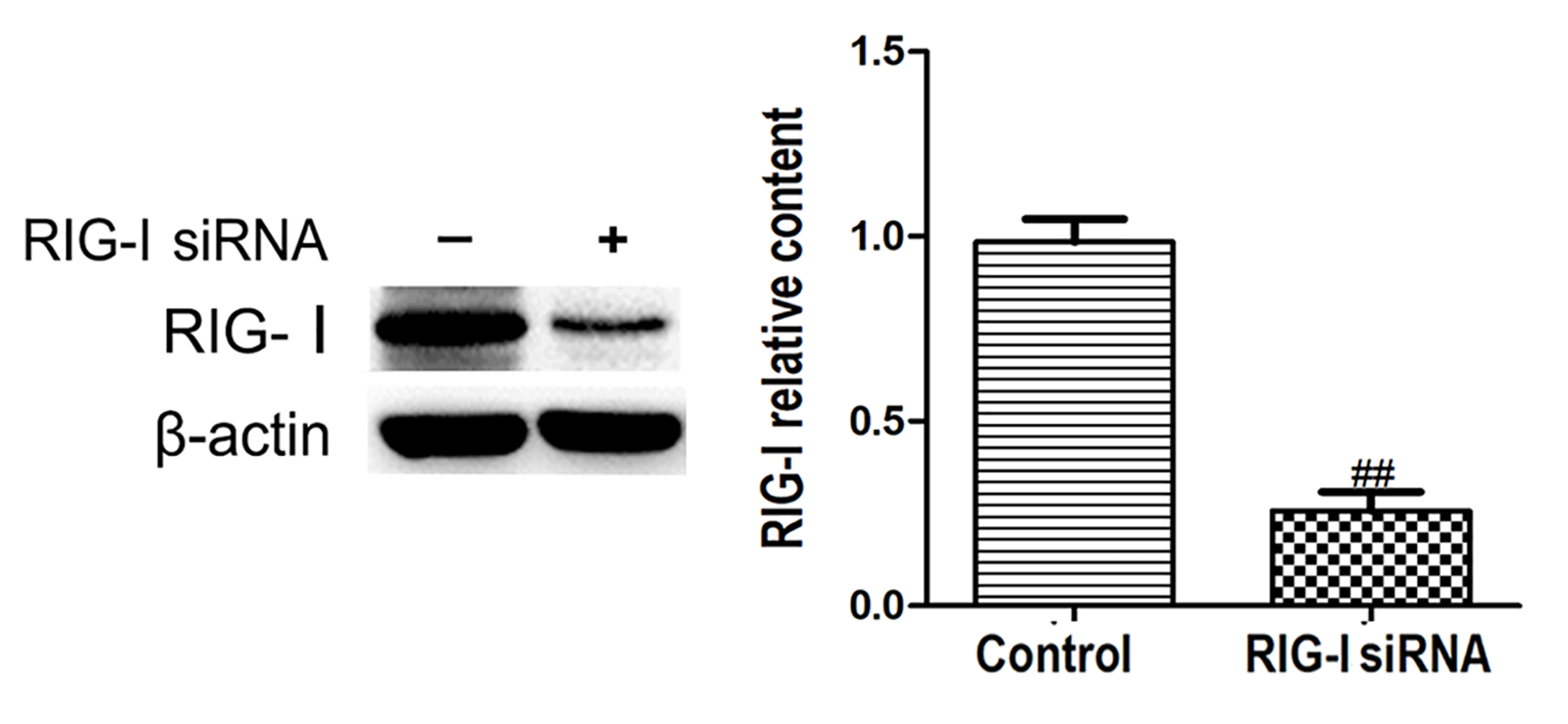

Supplement: Supplementary Materials — 1. The chemical identification of Panax notoginseng saponins. Panax notoginseng saponins (PNS)is the total saponins extracted from the main roots or rhizomes of Panax notoginseng (Burk.) F. H. Chen. PNS used in this study was a standard substance that purchased from the National Institutes for food and drug Control (NIFDC, China), which was identifiably composed of Notoginsenoside R1, ginsenoside Rg1, ginsenoside Re, ginsenoside Rb1, ginsenoside Rd by HPLC analysis (Fig S1). The chemical structure of each component was shown (Fig S2). 2. Transfection of siRNAs in BMECs. Short interfering RNA was used to silence RIG-I as the control, to further determine the pivotal role of RIG-I in ischemic injury. Transfection was performed by culturing the BMECs in siRNA Transfection Medium containing the siRNA Transfection Reagent mixture according to the manufacturer's protocol. Blank siRNA was used as a control. The expression of RIG-I was detected by Western Blotting to evaluate the efficiency of the transfection. As shown in Figure S3, the expression of RIG-I protein in the RIG-I siRNA transfection was significantly lower than control group (p < 0.01), suggesting that the transfection was successful and efficient. [file 8878428.f1.zip › figure s3.tif]
